# Childhood Obesity-Related Mechanisms: MicroRNome and Transcriptome Changes in a Nested Case-Control Study

**DOI:** 10.3390/biomedicines9080878

**Published:** 2021-07-23

**Authors:** Jin Hee Kim, Da Hae Kim, Youn-Hee Lim, Choong Ho Shin, Young Ah Lee, Bung-Nyun Kim, Johanna Inhyang Kim, Yun-Chul Hong

**Affiliations:** 1 Department of Integrative Bioscience & Biotechnology, Sejong University, 209 Neungdong-ro, Gwangjin-gu, Seoul 05006, Korea; dahae0218@sju.ac.kr; 2 Institute of Environmental Medicine, Seoul National University Medical Research Center, Seoul 03080, Korea; younhee.lim@sund.ku.dk; 3 Environmental Health Center, Seoul National University College of Medicine, Seoul 03080, Korea; 4 Department of Pediatrics, Seoul National University College of Medicine, Seoul 03080, Korea; chshinpd@snu.ac.kr (C.H.S.); nina337@snu.ac.kr (Y.A.L.); 5 Division of Children and Adolescent Psychiatry, Department of Psychiatry, Seoul National University Hospital, Seoul 03080, Korea; kbn1@snu.ac.kr; 6 Department of Psychiatry, Hanyang University Medical Center, Seoul 04763, Korea; iambabyvox@hanmail.net; 7 Department of Preventive Medicine, Seoul National University College of Medicine, Seoul 03080, Korea

**Keywords:** children, obesity, miRNA, mRNA, omics, pathway, disease class

## Abstract

Childhood obesity could contribute to adulthood obesity, leading to adverse health outcomes in adults. However, the mechanisms for how obesity is developed are still unclear. To determine the epigenome-wide and genome-wide expression changes related with childhood obesity, we compared microRNome and transcriptome levels as well as leptin protein levels in whole bloods of 12 obese and 24 normal children aged 6 years. miR-328-3p, miR-1301-3p, miR-4685-3p, and miR-6803-3p were negatively associated with all obesity indicators. The four miRNAs were also associated with 3948 mRNAs, and separate 475 mRNAs (185 among 3948 mRNAs) were associated with all obesity indicators. The 2533 mRNAs (64.2%) among the 3948 mRNAs and 286 mRNAs (60.2%) among the 475 mRNAs were confirmed as targets of the four miRNAs in public databases through miRWalk 2.0. Leptin protein was associated with miR-6803-3p negatively and all obesity indicators positively. Using DAVID bioinformatics resources 6.8, top three pathways for obesity-related gene set were metabolic pathways, pathways in cancer, and PI3K-Akt signaling pathway. The top three obesity-related disease classes were metabolic, cardiovascular, and chemdependency. Our results support that childhood obesity could be developed through miRNAs-related epigenetic mechanism and, further, these obesity-related epigenetic changes could control the pathways related with the development of various diseases.

## 1. Introduction

Childhood obesity could contribute to adulthood obesity, leading to adverse health outcomes in adults including diabetes and hypertension [1,2]. Because childhood obesity has been steadily growing worldwide, it has become an important health issue in childhood worldwide, including in Korean children [3]. Although dietary intake and exercise are known to be major factors for childhood obesity, limited evidence has been reported for the mechanisms of how childhood obesity could be developed [4]. Recently, several studies have suggested that epigenetic changes in early life could be important in the development of diseases [5,6,7]. Therefore, exploring obesity-related epigenome-wide expression changes in young children may be very important for the identification of the obesity-related pathway in children, and the prevention and treatment of childhood obesity-related diseases.

MicroRNAs (miRNAs) are a class of small (~22 nucleotide) endogenous non-coding RNAs [8]. Because miRNAs could regulate gene expression post-transcriptionally by binding to mRNAs, inhibiting translation, and promoting mRNA degradation, those could affect physiological and pathological processes in relation with a variety of diseases [9,10]. Several studies have shown that miRNAs could be up- or down-regulated in relation with obesity [9,11]. However, miRNAs reported to be regulated in relation with childhood obesity were inconsistent depending on the studies [12].

For a long time, the function of a gene was thought to be completed through its mRNA and protein, based on the central dogma [13]. For this reason, many efforts to find molecular markers based on the mRNAs and proteins have been conducted, including changes of mRNA related with chemical exposures [14,15]. Genome-wide mRNA expressions (transcriptome) have been also broadly used to explore fundamental mechanisms of target chemicals [14]. In fact, several studies have shown that a variety of mRNAs could be up- or down-regulated in relation with obesity [16]. Therefore, identification of mRNA markers changing in relation with obesity may be important for prevention and tailored therapy of obesity-related diseases. However, limited evidence was available for transcriptome change related with childhood obesity [16]. Furthermore, there were no reports for transcriptome change in relation with epigenome-wide miRNAs (microRNome).

Therefore, in the present study, we conducted a nested case-control study using the Environment and Development of Children (EDC) cohort. To evaluate the relationship among miRNA expressions, mRNA expressions, and childhood obesity, we conducted microRNome and transcriptome analyses in obese children and age- and sex-matched normal controls, and investigated how miRNAs and mRNAs could be related with childhood obesity. We also explored for functional pathways and disease classes related with childhood obesity in relation with miRNAs and mRNAs.

## 2. Materials and Methods

### 2.1. Study Participants and Blood Sampling

A total of 560 children among 726 children enrolled in the EDC cohort [17] visited health examination center in Seoul National University Hospital at 6 years old. Whole blood samples of subjects were collected for RNA extraction in the PAXgene Blood RNA Tube (PreAnalytiX, Hombrechtikon, Switzerland) containing a proprietary reagent for stabilization of intracellular RNA immediately upon collection since April 2016. However, all data including basic characteristics and biospecimens collected since January 2017 were not be opened to be used at the time we started the present study in accordance with the scientific research committee policy for the EDC cohort. Therefore, only 238 children at 6 years old, who visited the EDC center between April 2016 and December 2016, provided whole blood for total RNA extraction, and did not have any chronic diseases or abnormal values for urinary uric acid and creatinine levels were targeted in the present study. We selected obese cases and normal controls among the 238 children based on the national pediatric obesity criteria for Korean children at pediatric age ≥2 years old. After calculation of z-score for body mass index (BMI, kg/m^2^), children with BMI z-score ≥85th percentile and <95th percentile were diagnosed as over-weighted, children with BMI z-score ≥95th percentile and <99th percentile as obese, and children with BMI z-score ≥99th percentile as extremely obese [18]. Since we had small number of obese or extremely obese children, we selected 13 children with BMI z-score ≥85th percentile as obese cases and 26 children with BMI z-score <85th percentile as normal controls matched with the cases for sex and age (months) (1 case vs. 2 controls). The total RNA for microRNome and transcriptome level measurements was extracted from 39 blood samples obtained from 13 obese cases and 26 normal controls. However, since the concentration of total RNA obtained from one case among 13 cases was too low to conduct array hybridization, total RNA samples obtained from the case and the case-matched 2 controls were not used for array hybridization, and finally 36 RNA samples obtained from 12 cases and their matched 24 controls were used for microRNome and transcriptome level measurements. For all cases and controls, we also measured two obesity-related protein markers, adiponectin and leptin, using serum separated from blood samples collected in a SST tube (BD Vacutainer^®^ SST™ Blood Collection Tube, BD, Breda, The Netherlands).

### 2.2. Total RNA Extraction and Quality Assurance

Total RNA was extracted from blood samples collected from children using the Blood miRNA Kit (PreAnalytiX, Hombrechtikon, Switzerland). The purity and quantity of total RNA were evaluated using the ND-1000 Spectrophotometer (NanoDrop, Wilmington, NC, USA). Absorbance measurements at 260 nm are commonly used to quantify RNA. The RNA sample was considered to be relatively pure if the ratio of absorbance at 260 nm to absorbance at 280 nm or 230 nm was ranged from 1.7 to 2.0. RNA integrity was evaluated based on an RNA Integrity Number (RIN) value greater than or equal to 8 using the Agilent 2100 Bioanalyzer (Agilent Technologies, Palo Alto, CA, USA).

### 2.3. MicroRNome Level Measurements and Data Filtering

MicroRNome levels were measured using the Affymetrix GeneChip^®^ miRNA 4.0 array (Santa Clara, CA, USA) according to the manufacturer’s protocol. Briefly, 130 ng of total RNA were labeled with the FlashTag™ Biotin HSR RNA Labeling Kit (Life Technologies, Carlsbad, CA, USA). The labeled RNA was heated to 99 °C for 5 min and then to 45 °C for 5 min. All hybridization, washing, staining, and scanning were conducted following the protocol produced with the GeneChip™ Expression Wash, Stain and Scan (Affymetrix, Santa Clara, CA, USA). Shortly, RNA-array hybridization was performed for 16 h at 48 °C with agitation at 60 rotations per minute on an GeneChip^®^ Hybridizaiton oven (Affymetrix, Santa Clara, CA, USA). The chips were washed and stained using a GeneChip^®^ Fluidics Station 450 (Affymetrix, Santa Clara, CA, USA) and then scanned with an GeneChip^®^ GCS 3000 scanner (Affymetrix, Santa Clara, CA, USA) to detect the miRNA levels. Signal values were computed using the Affymetrix^®^ GeneChip™ Command Console (AGCC) software (Santa Clara, CA, USA) for raw data extraction of miRNAs according to the Affymetrix data extraction protocol (https://tools.thermofisher.com/content/sfs/manuals/agcc_command_console_user_guide.pdf accessed on 1 July 2020). Descriptive summarization for miRNA levels including normalization was conducted using Affymetrix^®^ Power Tools (APT) software apt-2.10.2.2 (Santa Clara, CA, USA) (affymetrix-power-tools.html accessed on 1 Jul, 2020) and all array data were filtered based on Homo sapiens. Only miRNAs that passed for all samples based on the default values were used in the statistical analyses.

### 2.4. Transcriptome Level Measurements and Data Filtering

Transcriptome levels were measured using the Affymetrix GeneChip^®^ Human Gene 2.0 ST Array (Santa Clara, CA, USA) according to the manufacturer’s protocol. Briefly, 100 ng of total RNA was used for cDNA synthesis using the GeneChip Whole Transcript (WT) Amplification kit (GeneChip Whole Transcript PLUS reagent Kit, Santa Clara, CA, USA). The sense cDNA was then fragmented and biotin-labeled with terminal deoxynucleotidyl transferase (TdT) using the GeneChip WT Terminal labeling kit (GeneChip Whole Transcript PLUS reagent Kit, Santa Clara, CA, USA). Approximately 5.5 μg of labeled DNA was hybridized to the Affymetrix GeneChip^®^ Human Gene 2.0 ST Array at 45 °C for 16 h and then washed and stained using a GeneChip^®^ Fluidics Station 450, then scanned with a GeneChip^®^ GCS 3000 scanner to detect the mRNA levels. Signal values were computed using the Affymetrix^®^ GeneChip™ Command Console (AGCC) software (Santa Clara, CA, USA) for raw data extraction of mRNAs according to the Affymetrix data extraction protocol (https://tools.thermofisher.com/content/sfs/manuals/agcc_command_console_user_guide.pdf accessed on 1 July 2020). Descriptive summarization for mRNA levels including normalization was conducted using Affymetrix^®^ Power Tools (APT) software apt-2.10.0 (Santa Clara, CA, USA) (affymetrix-power-tools.html accessed on 22 June 2020) and all array data were filtered based on Homo sapiens.

### 2.5. Measurement of Adiponectin and Leptin Levels in Blood

Because our target disease was obesity, we measured blood levels of two obesity-related proteins, adiponectin and leptin.

Adiponectin level in serum was measured using BioVendor’s Human Adiponectin ELISA system (BioVendor, Modrice, Czech Republic) by manufacturer’s recommendation. In brief, 50 μL of diluted sample was incubated in wells coated with recombinant adiponectin together with 50 μL of horse radish peroxidase-labelled anti-adiponectin antibody solution for 2 h with shaking. After a thorough wash, 200 μL of hydrogen peroxide/TMB substrate solution was added and incubated at room temperature for 10 min. After the reaction is stopped by addition of sulfuric acid solution, absorbance of the resulting yellow-colored product is measured spectrophotometrically at 450 nm. The absorbance is inversely proportional to the adiponectin concentration.

Leptin level in serum was also measured using a Human Leptin RIA Kit (Millipore, Saint Louis, MO, USA) with radioimmunoassay system by manufacturer’s recommendation. In brief, 100 μL of sample was vigorously mixed with 100 μL of Assay Buffer, 100 μL of ^125^I-Human Leptin, and 100 μL of Human Leptin antibody and incubated for 20 h at 4 °C. After mixture was vigorously mixed with 1 mL of cold Precipitating Reagent and incubated at 4 °C for 20 min, the mixture was centrifuged at 4 °C and supernate was drained. Leptin protein level was counted with pellets using an automated gamma counter possessing data reduction capabilities.

### 2.6. Statistical Analyses

Basic characteristics were compared between cases and controls using χ^2^-test for frequency comparison and using t-test for mean comparison. Only for miRNAs passed for all subjects, the relationships among miRNAs, mRNAs, obesity-related proteins, and three obesity indicators were evaluated. Relations among miRNAs, mRNAs, obesity-related proteins, and two obesity indicators (BMI and BMI z-score) were evaluated using linear regression, and the relations of miRNAs, mRNAs, and obesity-related proteins with obesity development were evaluated using logistic regression. In all models, we adjusted for sex, age (months), calorie intake, and current diseases. All statistical analyses for differential expressions and visualizations were conducted using SAS Enterprise 7.1 (SAS Institute Inc., Cary, NC, USA) and R statistical language 3.6.3 (https://www.r-project.org/ accessed on 1 July 2020).

### 2.7. Target Gene Prediction of Obesity-Associated miRNAs and Comparison with Obesity-Related mRNAs

For miRNAs statistically associated with all three obesity indicators (BMI, BMI z-score, and obesity development) (named as obesity-associated miRNAs), we predicted target genes of obesity-associated miRNAs using publicly used target prediction programs. The set of obesity-associated miRNAs was uploaded on miRWalk 2.0, a comprehensive atlas of predicted and validated miRNA-target interactions (http://zmf.umm.uni-heidelberg.de/apps/zmf/mirwalk2/custom.html accessed on 1 March 2021), and then mRNA targets were selected if those were significantly predicted or validated in at least one of 12 programs on miRWalk 2.0, including miRWalk, MicroT4, miRanda, miRBridge, miRDB, miRMap, miRNAMap, PICTAR2, PITA, RNA22, RNAhybird, and Targetscan based on *p* < 0.05. The predicted target genes were also compared with mRNAs which were significantly associated with obesity-associated miRNAs (named as miRNAs-associated mRNAs) and obesity-associated mRNAs.

### 2.8. Exploring Networking among miRNAs, mRNAs, Obesity-Related Proteins, and Obesity using Cytoscape

Networking among miRNAs, mRNAs, obesity-related proteins, and obesity was explored using the Cytoscape version 3.8.0 (U.S. National Institute of General Medical Sciences (NIGMS), Bethesda, MD, USA). We assigned miRNAs, mRNAs, obesity-related proteins, and obesity indicators as nodes, and directions for statistical associations among the nodes as edges. Because our outcome was obesity, we assigned obesity node as a value 1 with full red color and then calculated values for miRNA (or mRNA) nodes as 1 divided by β values representing sizes of statistical associations between miRNAs (or mRNAs) and BMI (or BMI z-score). If there were absolute values for miRNA (or mRNA) nodes over 1, we divided the values for each miRNA (or mRNA) node by maximum absolute value for miRNA (or mRNA) nodes to make the miRNA (or mRNA) nodes ranged from −1 to 1. Because odds ratio (OR) > 1 and OR < 1, respectively, mean increase and decrease for risk of obesity development, we calculated miRNA (or mRNA) node values as log_10_ OR divided by maximum absolute level of log_10_ OR to make obesity node as a value 1. Node values for proteins were fixed as 1. The color for all nodes was represented as red if it increased on obesity and blue if it decreased on obesity, while the color for edges was represented as red for positive association and blue for negative association. In addition, considering the coinstantaneous association of each mRNA with multiple miRNAs, we grouped mRNAs which were associated with specific miRNAs.

We also validated the networking based on STRING, protein-protein interaction networks, providing information of interactions among proteins (https://string-db.org/ accessed on 1 March 2021).

### 2.9. Exploring Functional Pathways and Diseases Classes

We explored functional pathways and diseases classes related with obesity using the Database for Annotation, Visualization and Integrated Discovery (DAVID) 6.8 Beta Knowledgebase (https://david-d.ncifcrf.gov/ accessed on 1 March 2021). After uploading the gene sets on DAVID, we explored the Kyoto Encyclopedia of Genes and Genomes (KEGG) pathways and Genetic Association Database (GAD) disease classes related with obesity.

## 3. Results

### 3.1. Study Population

Our participants were composed of 18 boys and 18 girls (Table 1). Because obese cases were matched with normal controls for sex and age (months), mean age was 70.1 months in both cases and controls (Table 1). Children’ BMI (as well as weight) was significantly different between obese cases and normal controls (*p* < 0.0001), but parental BMIs were not different between cases and controls (Table 1). Because physical activity and calorie intake could affect obesity in children, we compared physical activity and calorie intake between obese cases and normal controls as well. Although we found no significant differences, a little less physical activity and a little higher calorie intake per day in obese cases were found compared with those of normal controls (Table 1). We also compared blood levels of adiponectin and leptin as obesity-related proteins between obese cases and normal controls. Leptin level was significantly different between cases and controls (*p* = 0.0373), but adiponectin was not different between them (Table 1).

### 3.2. Relations among miRNAs, mRNAs, and Obesity Indicators

**Figure 1:**
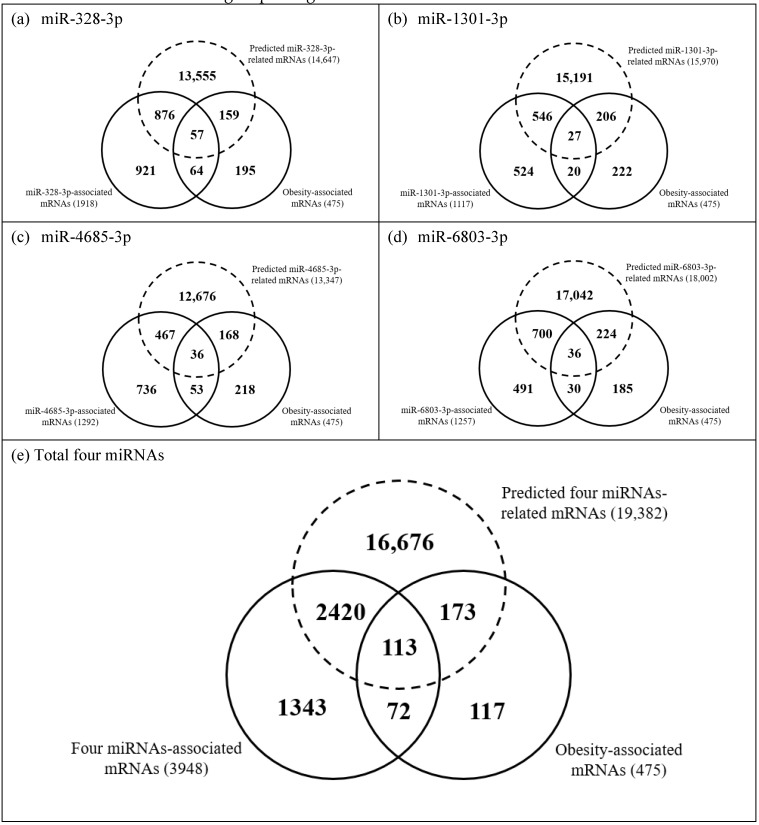
Venn diagrams comparing counts of associations among 3948 mRNAs statistically associated with four obesity-associated miRNAs, 19,382 genes predicted as targets of the four obesity-associated miRNAs, and 475 mRNAs statistically associated with three obesity indicators. (**a**) miR-328-3p (**b**) miR-1301-3p (**c**) miR-4685-3p (**d**) miR-6803-3p (**e**) Total four miRNAs.

The miRNAs statistically associated with three obesity indicators are listed in Table 2. Four miRNAs, miR-328-3p, miR-1301-3p, miR-4685-3p, and miR-6803-3p, showed statistically negative associations with all three obesity indicators, BMI, BMI z-score, and obesity development (*p* < 0.05 for all relations) (Table 2). The four obesity-associated miRNAs (miR-328-3p, miR-1301-3p, miR-4685-3p, and miR-6803-3p) were also found to be statistically associated with 3948 mRNAs (*p* < 0.05 for all relations) (Table S1). Further, a separate 475 mRNAs were statistically associated with all three obesity indicators (*p* < 0.05 for all relations) (Table S2). Therefore, we compared 3948 mRNAs in Table S1 with 475 mRNAs in Table S2 (Figure 1). Among 3948 four miRNAs-associated mRNAs, only 185 mRNAs were found to be statistically associated with all three obesity indicators (Figure 1). In more detail, miR-328-3p, miR-1301-3p, miR-4685-3p, and miR-6803-3p, respectively, were consistently associated with 121 mRNAs, 47 mRNAs, 89 mRNAs, and 66 mRNAs among the 475 obesity-associated mRNAs (Figure 1).

### 3.3. Prediction for Target Genes of the Four Obesity-Associated miRNAs and Comparison with Four miRNAs-Associated mRNAs or Obesity-Associated mRNAs

In the prediction on target genes of four miRNAs, the number of target genes of miR-328-3p, miR-1301-3p, miR-4685-3p, and miR-6803-3p were found to be 14,647, 15,970, 13,347, and 18,002, respectively (Figure 1 and Table S3). Totally, 2533 mRNAs among the 3948 four miRNAs-associated mRNAs and 286 mRNAs among the 475 obesity-associated mRNAs were confirmed as predicted target genes (Figure 1). The gene sets for each mRNAs group in Figure 1 were listed in Table S4.

### 3.4. Relations among miRNAs, mRNAs, and Obesity with Leptin

**Figure 2:**
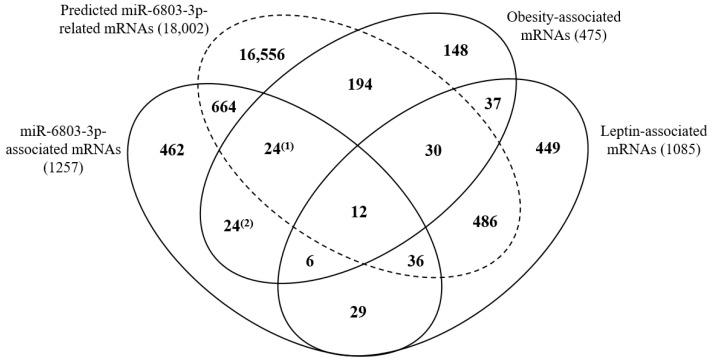
Venn diagrams comparing counts of associations between mRNAs in Figure 1 and leptin protein-associated mRNAs. Superscripted (1) and (2) means different groups for same counts.

Because we found differentially expressed leptin levels between obese cases and normal controls, we evaluated the relations among the four obesity-associated miRNAs, obesity-associated mRNAs, and obesity indicators with leptin protein. Only one miRNA, miR-6803-3p, showed a statistically negative association with leptin, while leptin was associated with all three obesity indicators (*p* < 0.05 for all relations) (Table 3). On the other hand, 1085 mRNAs were statistically associated with leptin with 588 positive and 497 negative associations (Figure 2 and Tables S5 and S6) (*p* < 0.05 for all).

Only 33 mRNAs among 185 mRNAs overlapped between the 3945 four miRNAs-associated mRNAs and the 475 obesity-associated mRNAs were statistically associated with leptin with 21 positive and 11 negative associations (*p* < 0.05 for all). Particularly for miR-6803-3p, 18 mRNAs were associated with leptin with 9 positive and 9 negative associations (Figure 2). Only 12 mRNAs among the 18 mRNAs were confirmed in both statistical analyses and target prediction and those genes were as follows; *CDC-like kinase 2* (*CLK2*), *DEAD-box helicase 41* (*DDX41*), *glutamate rich 6* (*ERICH6*), *leucine rich repeats and calponin homology domain containing 2* (*LRCH2*), *progestin and adipoQ receptor family member 7* (*PAQR7*), *pyruvate dehydrogenase complex component X* (*PDHX*), *piwi-like RNA-mediated gene silencing 1* (*PIWIL1*), *RAB3C, member RAS oncogene family* (*RAB3C*), *SSX family member 5* (*SSX5*), *transcription factor 15* (*TCF15*), *tetratricopeptide repeat domain 32* (*TTC32*), and *zinc finger protein 362* (*ZNF362*).

### 3.5. Networking among miRNAs, mRNAs, and Obesity with Leptin

**Figure 3:**
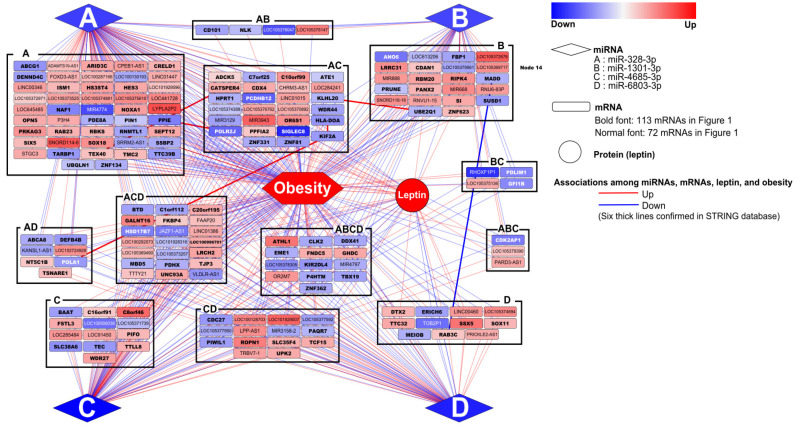
Networking among four obesity-associated miRNAs, 185 mRNAs which were statistically associated with four miRNAs and three obesity indicators, and obesity representing BMI z-score with leptin using Cytoscape.

We mapped networking among the four obesity-associated miRNAs, 185 mRNAs which were statistically associated with all four miRNAs and three obesity indicators, and obesity representing BMI z-score with leptin using Cytoscape (Figure 3). We found complicated, but consistent networking each other statistically. The trend of relations among those was replicated for obesity representing BMI or obesity development as well (Figures S1 and S2).

We validated functional networking found in our study based on STRING providing information of interactions among proteins. We confirmed six interactions among proteins, which were statistically associated with each other at mRNA levels (β = −0.39 and *p* = 0.0013 for MAP kinase activating death domain (MADD) and RAB3C, β = 0.60 and *p* = 0.0009 for ubiquitin conjugating enzyme E2 Q1 (UBE2Q1) and kelch-like family member 20 (KLHL20), β = 0.39 and *p* = 0.0327 for kinesin family member 2A (KIF2A) and major histocompatibility complex, class II, DO alpha (HLA-DOA), β = 0.57 and *p* < 0.0001 for DNA polymerase alpha 1, catalytic subunit (POLA1) and KIF2A, β = 0.40 and *p* = 0.0056 for RNA polymerase II subunit J (POLR2J) and peptidylprolyl isomerase E (PPIE), and β = 0.29 and *p* = 0.0292 for cation channel sperm associated 4 (CATSPER4) and catsper channel auxiliary subunit zeta (CATSPERZ, also called TEX40).

### 3.6. KEGG Pathways and GAD Disease Classes Related with Obesity

We pooled the 3948 four miRNAs-associated mRNAs, the 19,382 predicted four miRNAs-related mRNAs, and the 475 obesity-associated mRNAs in Figure 1 and then explored functional pathways and disease classes for the full genes set (*n* = 20,914) using DAVID 6.8 system.

Totally, top five obesity-related KEGG pathways were metabolic pathways (hsa01100), pathways in cancer (hsa05200), PI3K-Akt signaling pathway (hsa04151), neuroactive ligand-receptor interaction (hsa04080), and MAPK signaling pathway (hsa04010) in order of precedence (Table 4), while top five obesity-related GAD disease classes were metabolic, cardiovascular, chemdependency, immune, and neurological in order of precedence (Table 5).

## 4. Discussion

In the present study, we explored microRNome and transcriptome changes related with childhood obesity. Four miRNAs, miR-328-3p, miR-1301-3p, miR-4685-3p, and miR-6803-3p, were negatively associated with three obesity indicators. The four miRNAs were associated with 3948 mRNAs and separate 475 mRNAs were associated with three obesity indicators. The 2533 mRNAs (64.2%) among 3948 mRNAs and 286 mRNAs (60.2%) among 475 mRNAs were confirmed in public databases through the target prediction programs. The 185 mRNAs that overlapped between the 3948 mRNAs and the 475 mRNAs showed consistently significant associations with three obesity indicators. Furthermore, leptin protein was associated with obesity indicators positively and with miR-6803-3p negatively. We also explored KEGG pathways and GAD disease classes related with childhood obesity, and found that metabolic and cancer pathways and metabolic and cardiovascular disease classes were found to be strongly related with childhood obesity.

In the present study, we measured miRNA and mRNA levels using an array system and evaluated the relations among miRNAs, mRNAs, and obesity indicators. Even though the arrays used in our study contained almost of miRNAs and mRNAs, we tried to confirm obesity-related genes determined in our statistical analyses in public databases as well. In total, 2706 mRNAs (63.9%) among 4238 mRNAs (combined from the 3948 four miRNAs-associated mRNAs and the 475 obesity-associated mRNAs) identified in our statistical analyses were confirmed in public databases, indicating that our results may be very reliable. Moreover, the remaining 1532 mRNAs were newly identified in our study. We also checked how much proportion among 19,382 mRNAs predicted as targets in our study were contained in our array system and found that 15,304 mRNAs (91.8%) among 16,676 mRNAs which were only predicted as targets, were contained in our array system, while 1372 mRNAs (8.2%) were not contained in our array system. Therefore, the 1372 mRNAs which were not contained in our array system need to be examined in the future as well.

Leptin is a hormone that restrains hunger and thus regulates energy balance [19]. Because we found significantly higher leptin protein levels in obese cases compared with normal controls, we also tried to evaluate the relations among the four miRNAs, mRNAs, and obesity with leptin. We found a negative association between miR-6803-3p and leptin levels and positive association between leptin and obesity indicators. Thus, we further evaluated mRNAs overlapped among miR-6803-3p-associated mRNAs, predicted miR-6803-3p-related mRNAs, leptin-associated mRNAs, and obesity-associated mRNAs. Particularly, based on 185 mRNAs which were consistently confirmed in statistical analyses as the four miRNAs- and obesity-associated mRNAs, we summarized networking among the four miRNAs, the 185 mRNAs, and obesity with leptin. We found complicated but consistent networking among those with statistically significant relationships. In particular, we found twelve genes related with leptin, which were confirmed in both statistical analyses and target prediction. All twelve genes participate in cell cycling or splicing; *CLK2* gene encodes a protein kinase that phosphorylates threonine/serine- or tyrosine-containing substrates and it regulates arginine- and serine-rich proteins of the spliceosome complex related with alternative splicing [20]. CLK2 promotes energy expenditure during intermittent fasting in mice on a high-fat diet [21]. Because this protein is necessary to maintain energy balance, chronic CLK2 inhibition in the hypothalamus was associated with an increase in the fasting blood glucose levels, while overexpressing CLK2 reversed the obese phenotype [22]. DDX41, DEAD-box protein with the conserved motif Asp-Glu-Ala-Asp (DEAD), is a putative RNA helicase involving alteration of RNA secondary structure related with translation initiation, nuclear and mitochondrial splicing, and ribosome and spliceosomal assembly [23]. Down-regulation of this protein could result in tumor cell growth [23]. *LRCH2* gene encodes a calponin homology domain-containing protein with leucine-rich repeat [24]. LRCH2 functions as a cytoskeletal scaffolding protein using its C-terminal calponin homology domain interacting with actin filaments [24]. *LRCH2* expression was related with tumorigenesis, and over-expression of the gene involved in several carcinogenesis, including low grade of melanomas and gliomas, while *LRCH2* gene expression was decreased in late stage breast cancer patients with metastasis [24]. PDHX protein is the component X of pyruvate dehydrogenase complex and it plays a role in energy production process because of its function converting pyruvate to acetyl coenzyme A in the mitochondrial matrix [25]. Because of its function on energy production, down-regulation of *PDHX* could result in low energy efficiency. In fact, defects on *PDHX* caused pyruvate dehydrogenase deficiency, resulting in lactic acidosis and neurological dysfunction in infancy and early childhood [26]. *PIWIL1* gene encodes a member of the PIWI subfamily of Argonaute proteins with conserved regions such as PAZ and Piwi motifs functioning in translational regulation including RNA silencing and self-renewal of germline and hematopoietic stem cells in human [27]. For these reasons, down-regulation of *PIWIL1* could affect our body to have a variety of problems including abnormal production of many blood cells. In fact, PIWIL1 was detected as a biomarker for a pre-metabolic syndrome state in a mouse model [28]. *RAB3C* gene encodes a small GTPase recycling phagocytosed major histocompatibility complex (MHC) class I to the cell surface [29]. Insulin-dependent co-localization of RAB3C and insulin-sensitive glucose transporter GLUT4 (Solute carrier family 2 member 4 (SLC2A4) named in human) in cardiac muscle of lean Zucker rats disappeared in obese Zucker rats [29], indicating a possible involvement of RAB3C in the pathogenesis of insulin resistance in obese animals. This abnormal localization could result in an abnormally increased expression of RAB3C to respond to insulin. SSX5 belongs to the synovial sarcoma X breakpoint proteins family and functions as transcriptional repressors eliciting spontaneous cellular and humoral immune responses in cancer patients, and thus it is potentially useful target in cancer vaccine-based immunotherapy [30]. TCF15 is a transcription factor and its dimerization with mesenchyme homeobox 2 induces fatty acid uptake in cardiac endothelial cells which are involved in various physiological processes including angiogenesis and the control of vasomotor tone related with blood flow [31]. However, the heterodimer also directly regulates the expression of CD36 molecule, which plays a role in atherosclerosis development [31,32,33]. However, the information for the other four out of twelve genes, *ERICH6*, *PAQR7*, *TTC32*, and *ZNF362*, was limited, although ERICH6 expression was restricted to testis, while PAQR7, TTC32, and ZNF362 are expressed in several tissues including kidney and testis (PAQR7), bone marrow and prostate (TTC32), and spleen and kidney (ZNF362) [34].

We also evaluated the relations among the four miRNAs, mRNA for leptin, and obesity indicators and found statistically significant and positive associations between mRNA for leptin and two obesity indicators, BMI and BMI z-score, with a marginal significance between mRNA for leptin and obesity development (data not shown here). However, we did not find any statistical association between miR-6803-3p and mRNA for leptin, with rather little increase of mRNA for leptin by up-regulation of miR-6803-3p (data not shown here). It could be plausible that, even though the level of mRNA for leptin did not change, protein leptin could be up-regulated by down-regulation of miR-6803-3p, if miR-6803-3p directly targets mRNA for leptin and thus inhibits translation of mRNA to leptin protein.

We validated functional networking found in our study based on STRING providing information of interactions among proteins. We confirmed 6 interactions among proteins, which were statistically associated with each other at mRNA levels, with a negative association between MADD and RAB3C and positive associations between UBE2Q1 and KLHL20, among KIF2A, HLA-DOA, and POLA1 (between KIF2A and HLA-DOA and between KIF2A and POLA1), between POLR2J and PPIE, and between CATSPER4 and TEX40. The statistical associations among those were supported by STRING, showing same directions consistent with associations obtained from our statistical analyses; Both MADD and RAB3C are related with proliferation. MADD interacts with TNF-alpha receptor 1 to activate mitogen-activated protein kinase (MAPK) and generate the apoptotic signal [35]. On the other hand, RAB3C is a member of the RAS oncogene family functioning as a small GTPase [36]. Down-regulation of MAPK and up-regulation of RAB3C in obese children could increase proliferation of cell by inhibiting apoptosis. UBE2Q1 and KLHL20 could be simultaneously regulated. Both proteins are involved in protein-protein interaction such as a function conjugating target proteins for degradation and cell signaling throughout the cell and extracellularly [37,38]. In particular, impairment of KLHL20-mediated autophagy regulation potentiates starvation-induced cell death and aggravates diabetes-associated myoatrophy [37]. Because these proteins are ubiquitously expressed in normal condition, down-regulation of these two proteins in obese children could induce abnormal condition in the body [37,38,39]. KIF2A plays for normal mitotic progression such as normal spindle activity during mitosis [40]. POLA1 is a catalytic subunit of DNA polymerase with an essential role in DNA replication which is necessary for mitosis like KIF2A [41]. HLA-DOA belongs to the HLA class II alpha chain paralogues and it participates in the regulation of HLA-DM-mediated peptide loading on MHC class II molecules [42]. It was recently reported to collaborate with kinesin family members including KIF2A for antigen cross-presentation in dendritic cells, specialized immune cells of lymph node [43]. For these reasons, loss of functions by down-regulation of KIF2A, POLA1, and HLA-DOA could result in unbalances of mitosis and immune system, which are playing roles as main systems recovering or protecting the body [41,43,44]. POLR2J is a subunit of RNA polymerase II, which is responsible for synthesizing mRNA in eukaryotes, and it was reported to interact with translation initiation factor eIF3 in addition of its involvement in RNA transcription [45]. PPIE is a member of the peptidyl-prolyl cis-trans isomerase family, which catalyze the cis-trans isomerization of proline imidic peptide bonds in oligopeptides and accelerate the folding of proteins [46]. Based on the functions of POLR2J and PPIE related with RNA transcription and protein folding, and even intervening steps between RNA transcription and protein translation, down-regulation of POLR2J and PPIE in our obese children could induce general problems related with functional protein deficiency in our body. Both *CATSPER4* and *TEX40* were up-regulated in obese children of our study. Both proteins were known to have restricted expression toward testis and involve in reproduction process by forming the voltage-gated calcium channel complex [47,48]. Thus, the barely restrained expression of the two proteins in other specimen, not in testis, in our children could induce additional problems. However, there was no functional evidences.

In the present study, we tried to find functional pathways and disease classes related with obesity for each genes set overlapped among the 3948 four miRNAs-associated mRNAs, the 19,382 predicted four miRNAs-related mRNAs, and the 475 obesity-associated mRNAs. However, we could not find specific trends for functional pathways and disease classes related with obesity in each set, because the number of genes overlapped was too small to find any statistical relation. Finally, we pooled the 3948 four miRNAs-associated mRNAs, the 19,382 predicted four miRNAs-related mRNAs, and the 475 obesity-associated mRNAs, and then explored functional pathways and disease classes for the full genes set. In this functional analyses, we found consistent KEGG pathways and GAD disease classes related with obesity, such as metabolic-related pathways or disease classes. Because our target disease was obesity, metabolic-related pathways or disease classes could be reasonable. In addition, we found that cancer-related pathways and cardiovascular or immune disease classes were related with childhood obesity. These pathways and disease classes could be plausible as well, with much evidence previously published [1,2,49,50,51,52]. miR-328-3p inhibits cell proliferation and carcinogenesis or metastasis in colon by inactivating cancer-related signaling pathways such as PI3K-Akt pathway [49], while PI3K/AKT pathway damaged in various tissues of the body leads to obesity [50], indicating that down-regulation of miR-328-3p found in obese cases could lead to cell proliferation and carcinogenesis. In addition, upregulation of insulin-like growth factor 1 receptor by degradation of miR-328-3p were observed in patients with idiopathic pulmonary arterial hypertension, which is a kind of cardiovascular disease [51]. The previous reports support our results that cardiovascular diseases class could be plausible as well because childhood obesity resulted in adult obesity, which is a main cause of cardiovascular diseases [1,2]. In other hands, ABHD11-AS1 upregulates STAT3 by sponging miR-1301-3p, resulting in promoted cell proliferation and metastasis similarly with miR-328-3p [52]. However, there was no evidence for miR-4685-3p and miR-6803-3p. Therefore, studies for the four obesity-associated miRNAs found in our study need to be further activated.

We also compared functional pathways and disease classes explored with the 19,382 predicted four miRNA-related mRNAs with those explored with another gene set (a newly identified genes set, *n* = 1532) excluding predicted four miRNA-related mRNAs from full genes set. When we used predicted four miRNA-related mRNAs set, we found the functional pathways and disease classes same with those of full genes set, while only two KEGG pathways, olfactory transduction (hsa04740) and microRNAs in cancer (hsa05206), were statistically significant for a newly identified genes set (data not shown here). Therefore, the 1532 mRNAs newly identified in our study need to be further studied.

In the present study, EDC cohort targeted young children at community level with blood collection from those young children, even though those children don’t have any specific disease. It makes very hard for us to recruit young participants at community level. Furthermore, we selected only children with high BMI z-score as obese cases in the cohort. For these reasons, we had a small sample size for our study and thus false positive error needs to be considered. However, we obtained consistent results in the present study, although we did not define cases and controls as children with more extreme BMI z-scores, e.g., children with BMI z-score ≥95th percentile as obese cases and children with BMI z-score <5th percentile as normal controls, and it could make a random error leading to a null hypothesis.

## 5. Conclusions

Childhood obesity could contribute to adulthood obesity, leading to adverse health outcomes in adults. However, the mechanisms for how childhood obesity is developed are still unclear. To efficiently halt this developing obesity-related diseases, it is important to understand the pathological process and offer early interventions. Our results suggest that childhood obesity could be developed through miRNAs-related epigenetic mechanisms and further the miRNAs-related epigenetic changes related with childhood obesity could also affect the development of various diseases other than obesity, including cardiovascular diseases and cancer. However, we should fill in the mechanistic gap focused on knowledge base from biological to medical transition in the future.

## Figures and Tables

**Table 1:** Characteristics of the participants.

Characteristic, Mean ± SE (Range) or no. (%)	Obese Children (*n* = 12)	Normal Children (*n* = 24)	*p*-Value ^1^
Child			
Age, months	70.1 ± 0.4 (69–73)	70.1 ± 0.3 (69–73)	1.0
Sex, no. of boys (%)	6 (50.0)	12 (50.0)	1.0
Weight, kg	25.6 ± 0.8 (21.5–30.5)	20.4 ± 0.4 (17.5–25.8)	<0.0001
Height, cm	118.5 ± 1.2 (112.3–125.1)	115.9 ± 0.6 (111.1–124.6)	0.0475
BMI, kg/m^2^	18.2 ± 0.3 (17.0–21.1)	15.1 ± 0.2 (13.7–17.0)	<0.0001
Vigorous physical activity, mins/day	32.5 ± 9.9 (0–120)	41.5 ± 6.1 (0–120)	0.4245
Calorie intake, kcal/day	1671.8 ± 129.5 (939.2–2646.4)	1462.4 ± 66.4 (853.9–2099.9)	0.1184
Current drug use, no. (%)			
No	12 (100)	22 (91.6)	0.5890
Flu	0 (0)	1 (4.2)	
Rhinitis	0 (0)	1 (4.2)	
Adiponectin, µg/mL	10.2 ± 0.7 (6.0–13.2)	9.0 ± 0.5 (5.5–17.6)	0.1744
Leptin, ng/mL	9.2 ± 1.5 (4.0–21.8)	5.7 ± 0.6 (3.0–15.6)	0.0373
Parent			
Mother BMI (before pregnancy), kg/m^2^	21.3 ± 0.7 (16.6–25.6)	21.1 ± 0.4 (18.1–25.4)	0.8159
Father BMI, kg/m^2^	25.3 ± 0.9 (19.6–29.8)	25.5 ± 0.6 (21.0–31.7)	0.8178

^1^ Student’s *t*-test for mean comparison and χ^2^ test for frequency comparison. BMI, body mass index; SE, standard error.

**Table 2:** Associations between four miRNAs levels and three obesity indicators.

			95% CI		
miRNA	Obesity Indicator	β or OR	Lower CI	Upper CI	*p*-Value
miR-328-3p	BMI	−1.83	−3.14	−0.52	0.0079
miR-1301-3p	BMI	−1.79	−3.51	−0.08	0.0412
miR-4685-3p	BMI	−1.49	−2.63	−0.35	0.0121
miR-6803-3p	BMI	−1.30	−2.25	−0.35	0.0091
miR-328-3p	BMI z-score	−1.04	−1.85	−0.22	0.0143
miR-1301-3p	BMI z-score	−1.08	−2.13	−0.03	0.0444
miR-4685-3p	BMI z-score	−0.89	−1.59	−0.19	0.0142
miR-6803-3p	BMI z-score	−0.78	−1.37	−0.20	0.0103
miR-328-3p	Obesity development	0.04	0.003	0.59	0.0192
miR-1301-3p	Obesity development	0.06	0.004	0.97	0.0475
miR-4685-3p	Obesity development	0.07	0.01	0.62	0.0178
miR-6803-3p	Obesity development	0.07	0.01	0.75	0.0289

β values for BMI and BMI z-score and OR for obesity development were evaluated after adjusted for sex, age (month), calorie intake, and current disease. BMI, body mass index; OR, odds ratio; CI, confidence interval.

**Table 3:** Relations of leptin protein with four miRNAs and three obesity indicators.

			95% CI		
Independent Variable	Dependent Variable	β or OR	Lower CI	Upper CI	*p*-Value
miR-328-3p	Leptin	−1.65	−4.80	1.50	0.2936
miR-1301-3p	Leptin	−0.88	−4.88	3.11	0.6540
miR-4685-3p	Leptin	−2.01	−4.65	0.64	0.1310
miR-6803-3p	Leptin	−2.26	−4.42	−0.10	0.0413
Leptin	BMI	0.28	0.15	0.42	0.0002
Leptin	BMI z-score	0.15	0.06	0.24	0.0020
Leptin	Obesity development	1.66	1.09	2.54	0.0180

β values for relations among miRNAs, leptin, and BMI or BMI z-score and OR for leptin and obesity development were evaluated after adjusted for sex, age (month), calorie intake, and current disease. BMI, body mass index; OR, odds ratio; CI, confidence interval.

**Table 4:** Top ten KEGG pathways related with obesity.

miRNA Name	KEGG Pathways	No. of Database-Matched Genes	No. of Pathway-Related Targets (%)	*p*-Value
miR-328-3p	hsa01100:Metabolic pathways	15,209	921 (6.1)	0.0030
	hsa05200:Pathways in cancer	15,209	348 (2.3)	<0.0001
	hsa04151:PI3K-Akt signaling pathway	15,209	281 (1.8)	0.0001
	hsa04010:MAPK signaling pathway	15,209	224 (1.5)	<0.0001
	hsa04080:Neuroactive ligand-receptor interaction	15,209	216 (1.4)	0.0231
	hsa04144:Endocytosis	15,209	213 (1.4)	<0.0001
	hsa05166:HTLV-I infection	15,209	211 (1.4)	0.0001
	hsa04014:Ras signaling pathway	15,209	199 (1.3)	<0.0001
	hsa04015:Rap1 signaling pathway	15,209	180 (1.2)	<0.0001
	hsa05205:Proteoglycans in cancer	15,209	179 (1.2)	<0.0001
miR-1301-3p	hsa01100:Metabolic pathways	16,121	980 (6.1)	0.0074
	hsa05200:Pathways in cancer	16,121	356 (2.2)	<0.0001
	hsa04151:PI3K-Akt signaling pathway	16,121	303 (1.9)	<0.0001
	hsa04010:MAPK signaling pathway	16,121	230 (1.4)	<0.0001
	hsa04080:Neuroactive ligand-receptor interaction	16,121	229 (1.4)	0.0316
	hsa04144:Endocytosis	16,121	222 (1.4)	<0.0001
	hsa05166:HTLV-I infection	16,121	220 (1.4)	0.0003
	hsa04014:Ras signaling pathway	16,121	205 (1.3)	<0.0001
	hsa04060:Cytokine-cytokine receptor interaction	16,121	201 (1.2)	0.0426
	hsa04510:Focal adhesion	16,121	192 (1.2)	<0.0001
miR-4685-3p	hsa05200:Pathways in cancer	13,811	320 (2.3)	<0.0001
	hsa04151:PI3K-Akt signaling pathway	13,811	260 (1.9)	0.0001
	hsa04010:MAPK signaling pathway	13,811	208 (1.5)	<0.0001
	hsa05166:HTLV-I infection	13,811	199 (1.4)	<0.0001
	hsa04144:Endocytosis	13,811	196 (1.4)	<0.0001
	hsa04014:Ras signaling pathway	13,811	186 (1.3)	<0.0001
	hsa04015:Rap1 signaling pathway	13,811	170 (1.2)	<0.0001
	hsa05205:Proteoglycans in cancer	13,811	169 (1.2)	<0.0001
	hsa04510:Focal adhesion	13,811	168 (1.2)	<0.0001
	hsa04024:cAMP signaling pathway	13,811	165 (1.2)	<0.0001
miR-6803-3p	hsa01100:Metabolic pathways	17,852	1076 (6.0)	0.0086
	hsa05200:Pathways in cancer	17,852	379 (2.1)	<0.0001
	hsa04151:PI3K-Akt signaling pathway	17,852	318 (1.8)	0.0004
	hsa04080:Neuroactive ligand-receptor interaction	17,852	256 (1.4)	0.0012
	hsa04010:MAPK signaling pathway	17,852	251 (1.4)	<0.0001
	hsa05166:HTLV-I infection	17,852	237 (1.3)	0.0004
	hsa04144:Endocytosis	17,852	228 (1.3)	<0.0001
	hsa04014:Ras signaling pathway	17,852	215 (1.2)	<0.0001
	hsa04015:Rap1 signaling pathway	17,852	204 (1.1)	<0.0001
	hsa04810:Regulation of actin cytoskeleton	17,852	202 (1.1)	<0.0001
Total	hsa01100:Metabolic pathways	19,922	1173 (5.9)	<0.0001
	hsa05200:Pathways in cancer	19,922	390 (2.0)	<0.0001
	hsa04151:PI3K-Akt signaling pathway	19,922	334 (1.7)	0.0016
	hsa04080:Neuroactive ligand-receptor interaction	19,922	272 (1.4)	0.0001
	hsa04010:MAPK signaling pathway	19,922	253 (1.3)	<0.0001
	hsa05166:HTLV-I infection	19,922	249 (1.2)	0.0004
	hsa04144:Endocytosis	19,922	237 (1.2)	0.0002
	hsa04014:Ras signaling pathway	19,922	221 (1.1)	0.0019
	hsa04810:Regulation of actin cytoskeleton	19,922	208 (1.0)	0.0001
	hsa04015:Rap1 signaling pathway	19,922	207 (1.0)	0.0004

**Table 5:** Top ten GAD disease classes related with obesity.

miRNA Name	GAD Disease Classes	No. of Database-Matched Genes	No. of Disease Class-Related Targets (%)	*p*-Value
miR-328-3p	METABOLIC	15,209	4014 (26.4)	<0.0001
	CARDIOVASCULAR	15,209	3286 (21.6)	<0.0001
	CHEMDEPENDENCY	15,209	2919 (19.2)	<0.0001
	NEUROLOGICAL	15,209	2218 (14.6)	0.0003
	PHARMACOGENOMIC	15,209	2178 (14.3)	<0.0001
	PSYCH	15,209	1573 (10.3)	<0.0001
	UNKNOWN	15,209	1253 (8.2)	0.0004
	OTHER	15,209	1242 (8.2)	0.0002
	DEVELOPMENTAL	15,209	1172 (7.7)	0.0002
	HEMATOLOGICAL	15,209	1169 (7.7)	0.0191
miR-1301-3p	METABOLIC	16,121	4422 (27.4)	<0.0001
	CARDIOVASCULAR	16,121	3565 (22.1)	<0.0001
	CHEMDEPENDENCY	16,121	3208 (19.9)	<0.0001
	NEUROLOGICAL	16,121	2396 (14.9)	<0.0001
	PHARMACOGENOMIC	16,121	2318 (14.4)	<0.0001
	INFECTION	16,121	1762 (10.9)	0.0304
	PSYCH	16,121	1703 (10.6)	<0.0001
	UNKNOWN	16,121	1356 (8.4)	<0.0001
	OTHER	16,121	1332 (8.3)	0.0003
	DEVELOPMENTAL	16,121	1290 (8.0)	<0.0001
miR-4685-3p	METABOLIC	13,811	3686 (26.7)	0.0001
	CARDIOVASCULAR	13,811	3017 (21.8)	<0.0001
	CHEMDEPENDENCY	13,811	2752 (19.9)	<0.0001
	NEUROLOGICAL	13,811	2051 (14.9)	0.0002
	PHARMACOGENOMIC	13,811	2006 (14.5)	<0.0001
	PSYCH	13,811	1448 (10.5)	<0.0001
	UNKNOWN	13,811	1145 (8.3)	0.0065
	OTHER	13,811	1129 (8.2)	0.0113
	DEVELOPMENTAL	13,811	1099 (8.0)	<0.0001
	HEMATOLOGICAL	13,811	1088 (7.9)	0.0046
miR-6803-3p	METABOLIC	17,852	4678 (26.2)	<0.0001
	CARDIOVASCULAR	17,852	3773 (21.1)	<0.0001
	CHEMDEPENDENCY	17,852	3340 (18.7)	<0.0001
	NEUROLOGICAL	17,852	2546 (14.3)	0.0069
	PHARMACOGENOMIC	17,852	2493 (14.0)	<0.0001
	PSYCH	17,852	1790 (10.0)	<0.0001
	UNKNOWN	17,852	1452 (8.1)	<0.0001
	DEVELOPMENTAL	17,852	1356 (7.6)	<0.0001
	HEMATOLOGICAL	17,852	1356 (7.6)	0.0074
	RENAL	17,852	1288 (7.2)	<0.0001
Total	METABOLIC	19,922	4959 (24.9)	<0.0001
	CARDIOVASCULAR	19,922	4003 (20.1)	<0.0001
	CHEMDEPENDENCY	19,922	3524 (17.7)	<0.0001
	IMMUNE	19,922	2786 (14.0)	0.0408
	NEUROLOGICAL	19,922	2701 (13.6)	0.0189
	PHARMACOGENOMIC	19,922	2650 (13.3)	<0.0001
	INFECTION	19,922	2057 (10.3)	<0.0001
	PSYCH	19,922	1880 (9.4)	<0.0001
	UNKNOWN	19,922	1556 (7.8)	<0.0001
	OTHER	19,922	1508 (7.6)	0.0006

## Data Availability

The datasets analyzed during the current study are not publicly available due to protection of participant confidentiality but are available from the corresponding author on reasonable request with assurances and plans in place to protect confidentiality.

## Supplementary Materials

The following are available online at https://www.mdpi.com/article/10.3390/biomedicines9080878/s1, Figure S1: Networking among four obesity-associated miRNAs, 185 mRNAs which were statistically associated with four miRNAs and three obesity indicators, and obesity representing BMI with leptin using Cytoscape, Figure S2: Networking among four obesity-associated miRNAs, 185 mRNAs which were statistically associated with four miRNAs and three obesity indicators, and obesity development with leptin using Cytoscape, Table S1: Associations between four miRNAs which were statistically associated with three obesity indicators and 3948 mRNAs, Table S2: Associations between 475 mRNA levels and three obesity indicators, Table S3: Predicted target genes of four obesity-associated miRNAs, Table S4: Gene sets in each group of Venn diagram in Figure 1, Table S5: Associations between 1085 mRNAs and leptin levels, Table S6: Gene sets in each group of Venn diagram in Figure 2.
